# UniRule: a unified rule resource for automatic annotation in the UniProt Knowledgebase

**DOI:** 10.1093/bioinformatics/btaa485

**Published:** 2020-05-12

**Authors:** Alistair MacDougall, Vladimir Volynkin, Rabie Saidi, Diego Poggioli, Hermann Zellner, Emma Hatton-Ellis, Vishal Joshi, Claire O’Donovan, Sandra Orchard, Andrea H Auchincloss, Delphine Baratin, Jerven Bolleman, Elisabeth Coudert, Edouard de Castro, Chantal Hulo, Patrick Masson, Ivo Pedruzzi, Catherine Rivoire, Cecilia Arighi, Qinghua Wang, Chuming Chen, Hongzhan Huang, John Garavelli, C R Vinayaka, Lai-Su Yeh, Darren A Natale, Kati Laiho, Maria-Jesus Martin, Alexandre Renaux, Klemens Pichler, The UniProt Consortium, Alex Bateman, Alan Bridge, Cathy Wu, Cecilia Arighi, Lionel Breuza, Elisabeth Coudert, Hongzhan Huang, Damien Lieberherr, Michele Magrane, Maria J Martin, Peter McGarvey, Darren Natale, Sandra Orchard, Ivo Pedruzzi, Sylvain Poux, Manuela Pruess, Shriya Raj, Nicole Redaschi, Lucila Aimo, Ghislaine Argoud-Puy, Andrea Auchincloss, Kristian Axelsen, Emmanuel Boutet, Emily Bowler, Ramona Britto, Hema Bye-A-Jee, Cristina Casals-Casas, Paul Denny, Anne Estreicher, Maria Livia Famiglietti, Marc Feuermann, John S Garavelli, Penelope Garmiri, Arnaud Gos, Nadine Gruaz, Emma Hatton-Ellis, Chantal Hulo, Nevila Hyka-Nouspikel, Florence Jungo, Kati Laiho, Philippe Le Mercier, Antonia Lock, Yvonne Lussi, Alistair MacDougall, Patrick Masson, Anne Morgat, Sandrine Pilbout, Lucille Pourcel, Catherine Rivoire, Karen Ross, Christian Sigrist, Elena Speretta, Shyamala Sundaram, Nidhi Tyagi, C R Vinayaka, Qinghua Wang, Kate Warner, Lai-Su Yeh, Rossana Zaru, Shadab Ahmed, Emanuele Alpi, Leslie Arminski, Parit Bansal, Delphine Baratin, Teresa Batista Neto, Jerven Bolleman, Chuming Chen, Yongxing Chen, Beatrice Cuche, Austra Cukura, Edouard De Castro, ThankGod Ebenezer, Elisabeth Gasteiger, Sebastien Gehant, Leonardo Gonzales, Abdulrahman Hussein, Alexandr Ignatchenko, Giuseppe Insana, Rizwan Ishtiaq, Vishal Joshi, Dushyanth Jyothi, Arnaud Kerhornou, Thierry Lombardot, Aurelian Luciani, Jie Luo, Mahdi Mahmoudy, Alok Mishra, Katie Moulang, Andrew Nightingale, Joseph Onwubiko, Monica Pozzato, Sangya Pundir, Guoying Qi, Daniel Rice, Rabie Saidi, Edward Turner, Preethi Vasudev, Yuqi Wang, Xavier Watkins, Hermann Zellner, Jian Zhang

**Affiliations:** European Molecular Biology Laboratory, European Bioinformatics Institute (EMBL-EBI), Wellcome Genome Campus, Hinxton, Cambridge CB10 1SD, UK; European Molecular Biology Laboratory, European Bioinformatics Institute (EMBL-EBI), Wellcome Genome Campus, Hinxton, Cambridge CB10 1SD, UK; European Molecular Biology Laboratory, European Bioinformatics Institute (EMBL-EBI), Wellcome Genome Campus, Hinxton, Cambridge CB10 1SD, UK; European Molecular Biology Laboratory, European Bioinformatics Institute (EMBL-EBI), Wellcome Genome Campus, Hinxton, Cambridge CB10 1SD, UK; Kantar Consulting, Casalecchio Di Reno, 40033 Bologna, Italy; European Molecular Biology Laboratory, European Bioinformatics Institute (EMBL-EBI), Wellcome Genome Campus, Hinxton, Cambridge CB10 1SD, UK; European Molecular Biology Laboratory, European Bioinformatics Institute (EMBL-EBI), Wellcome Genome Campus, Hinxton, Cambridge CB10 1SD, UK; European Molecular Biology Laboratory, European Bioinformatics Institute (EMBL-EBI), Wellcome Genome Campus, Hinxton, Cambridge CB10 1SD, UK; European Molecular Biology Laboratory, European Bioinformatics Institute (EMBL-EBI), Wellcome Genome Campus, Hinxton, Cambridge CB10 1SD, UK; European Molecular Biology Laboratory, European Bioinformatics Institute (EMBL-EBI), Wellcome Genome Campus, Hinxton, Cambridge CB10 1SD, UK; SIB Swiss Institute of Bioinformatics, Centre Medical Universitaire, CH-1211 Geneva 4, Switzerland; SIB Swiss Institute of Bioinformatics, Centre Medical Universitaire, CH-1211 Geneva 4, Switzerland; SIB Swiss Institute of Bioinformatics, Centre Medical Universitaire, CH-1211 Geneva 4, Switzerland; SIB Swiss Institute of Bioinformatics, Centre Medical Universitaire, CH-1211 Geneva 4, Switzerland; SIB Swiss Institute of Bioinformatics, Centre Medical Universitaire, CH-1211 Geneva 4, Switzerland; SIB Swiss Institute of Bioinformatics, Centre Medical Universitaire, CH-1211 Geneva 4, Switzerland; SIB Swiss Institute of Bioinformatics, Centre Medical Universitaire, CH-1211 Geneva 4, Switzerland; SIB Swiss Institute of Bioinformatics, Centre Medical Universitaire, CH-1211 Geneva 4, Switzerland; SIB Swiss Institute of Bioinformatics, Centre Medical Universitaire, CH-1211 Geneva 4, Switzerland; Protein Information Resource, University of Delaware, Newark, DE 19711, USA; Protein Information Resource, University of Delaware, Newark, DE 19711, USA; Protein Information Resource, University of Delaware, Newark, DE 19711, USA; Protein Information Resource, University of Delaware, Newark, DE 19711, USA; Protein Information Resource, University of Delaware, Newark, DE 19711, USA; Protein Information Resource, Georgetown University Medical Center, Washington, DC 20007, USA; Protein Information Resource, Georgetown University Medical Center, Washington, DC 20007, USA; Protein Information Resource, Georgetown University Medical Center, Washington, DC 20007, USA; Protein Information Resource, Georgetown University Medical Center, Washington, DC 20007, USA; European Molecular Biology Laboratory, European Bioinformatics Institute (EMBL-EBI), Wellcome Genome Campus, Hinxton, Cambridge CB10 1SD, UK; European Molecular Biology Laboratory, European Bioinformatics Institute (EMBL-EBI), Wellcome Genome Campus, Hinxton, Cambridge CB10 1SD, UK; European Molecular Biology Laboratory, European Bioinformatics Institute (EMBL-EBI), Wellcome Genome Campus, Hinxton, Cambridge CB10 1SD, UK; European Molecular Biology Laboratory, European Bioinformatics Institute (EMBL-EBI), Wellcome Genome Campus, Hinxton, Cambridge CB10 1SD, UK; SIB Swiss Institute of Bioinformatics, Centre Medical Universitaire, CH-1211 Geneva 4, Switzerland; Protein Information Resource, University of Delaware, Newark, DE 19711, USA

## Abstract

**Motivation:**

The number of protein records in the UniProt Knowledgebase (UniProtKB: https://www.uniprot.org) continues to grow rapidly as a result of genome sequencing and the prediction of protein-coding genes. Providing functional annotation for these proteins presents a significant and continuing challenge.

**Results:**

In response to this challenge, UniProt has developed a method of annotation, known as UniRule, based on expertly curated rules, which integrates related systems (RuleBase, HAMAP, PIRSR, PIRNR) developed by the members of the UniProt consortium. UniRule uses protein family signatures from InterPro, combined with taxonomic and other constraints, to select sets of reviewed proteins which have common functional properties supported by experimental evidence. This annotation is propagated to unreviewed records in UniProtKB that meet the same selection criteria, most of which do not have (and are never likely to have) experimentally verified functional annotation. Release 2020_01 of UniProtKB contains 6496 UniRule rules which provide annotation for 53 million proteins, accounting for 30% of the 178 million records in UniProtKB. UniRule provides scalable enrichment of annotation in UniProtKB.

**Availability and implementation:**

UniRule rules are integrated into UniProtKB and can be viewed at https://www.uniprot.org/unirule/. UniRule rules and the code required to run the rules, are publicly available for researchers who wish to annotate their own sequences. The implementation used to run the rules is known as UniFIRE and is available at https://gitlab.ebi.ac.uk/uniprot-public/unifire.

## 1 Introduction

The UniProt Knowledgebase (UniProtKB) is the main international resource providing open access to a database of protein sequences and function (The UniProt Consortium, 2019). UniProtKB is divided into two sections. One section (known as UniProtKB/Swiss-Prot) contains reviewed protein records and is carefully generated by experts who summarize the literature and organize that data into concise entries which are also machine readable. UniProtKB/Swiss-Prot release 2020_01 contains 562 119 protein records which have been reviewed and annotated in this way. The other section of UniProtKB (known as UniProtKB/TrEMBL) contains 178 million records in release 2020_01 for proteins which have not been expertly reviewed. Some of these records are for proteins which are the subject of experimental investigation. In due time, these will be expertly reviewed and transferred to UniProtKB/Swiss-Prot. However, most of the protein records in UniProtKB/TrEMBL are generated by prediction from genomic sequence data, with no prospect of their function being experimentally established. The sheer volume of genomic data arising from current sequencing projects means that the proportion of records in UniProtKB/TrEMBL representing predicted proteins predominates. The total number of protein records in UniProtKB/TrEMBL is expected to double every 18 months. A major challenge for UniProt is to provide annotation for these records that is accurate and detailed and resembles the annotation found in the reviewed records in UniProtKB/Swiss-Prot.

The UniProtKB/Swiss-Prot curated dataset is of huge value for computational biologists who train machine learning models on the data to infer the properties of newly predicted proteins (Fetrow and Babbitt, 2018; Rentzsch and Orengo, 2009). As a result, UniProtKB/Swiss-Prot data is utilized by a number of different protocols for protein function prediction (Schnoes *et al.*, 2009). In addition to making UniProtKB data publicly available to the research community, UniProt has created its own system that annotates experimentally uncharacterized proteins based on similarity to known experimentally characterized proteins. This system, UniRule, is a semi-automated rule-based computational annotation pipeline that integrates several rule systems [RuleBase, HAMAP (Pedruzzi *et al.*, 2015), PIRSR (Chen *et al.*, 2019) and PIRNR], thereby providing a single coordinated work flow for annotating unreviewed entries and increasing the quality and consistency of annotations for uncharacterized proteins. UniRule depends on the functional analysis of proteins carried out by InterPro, in which predictive models generated by HAMAP, PIRSR, PIRNR and other InterPro member databases, are used to predict the presence of functional domains and to assign protein sequences to protein families. In UniRule, the characterization of proteins by InterPro is combined with the detailed annotation found in the reviewed records in UniProtKB to create rules for propagating annotation to the unreviewed proteins in the database.

The UniRule system has been developed to provide up-to-date annotations within each UniProtKB release, which requires monitoring of both the underlying InterPro signatures (Mitchell *et al.*, 2019) and experimental information in UniProtKB/Swiss-Prot. The UniRule system provides annotations for around 30% (currently 53 million records) of the unreviewed UniProtKB/TrEMBL entries based on 6496 manually curated rules.

This article describes for the first time the UniRule annotation pipeline, what it is and how it works.

## 2 Materials and methods

### 2.1 Overview of UniRule database

The main aim of UniRule is to use InterPro family signatures as a basis for propagating annotation from reviewed to unreviewed protein records in UniProtKB. If the reviewed set of records in UniProtKB/Swiss-Prot that contain a particular protein family signature has consistent annotation, it is reasonable to use this protein signature to select unreviewed proteins in UniProtKB/TrEMBL and to transfer the annotation to them, assuming that they will fulfil the same biological role. In practice, UniRule rules contain several other conditions besides the protein family signature to define a consistently annotated set of reviewed records. The annotations in the rule are then applied to unreviewed records that satisfy the same set of conditions.

At each release of UniProtKB, every unreviewed UniProtKB/TrEMBL record is evaluated against every UniRule, and where the record meets the conditions of the rule, the associated annotations are added or updated as appropriate. The way rules are constructed allows quite complex condition requirements to be expressed within an individual rule, so that propagation of annotation can be subtly controlled for high accuracy and depth of annotation. Rules also incorporate fields for metadata which are not made publicly available (for instance: author, date created, date last modified) but are important for rule maintenance and update. The current list of rules is available on the UniProt website at https://www.uniprot.org/unirule/.

**Fig. 1.**
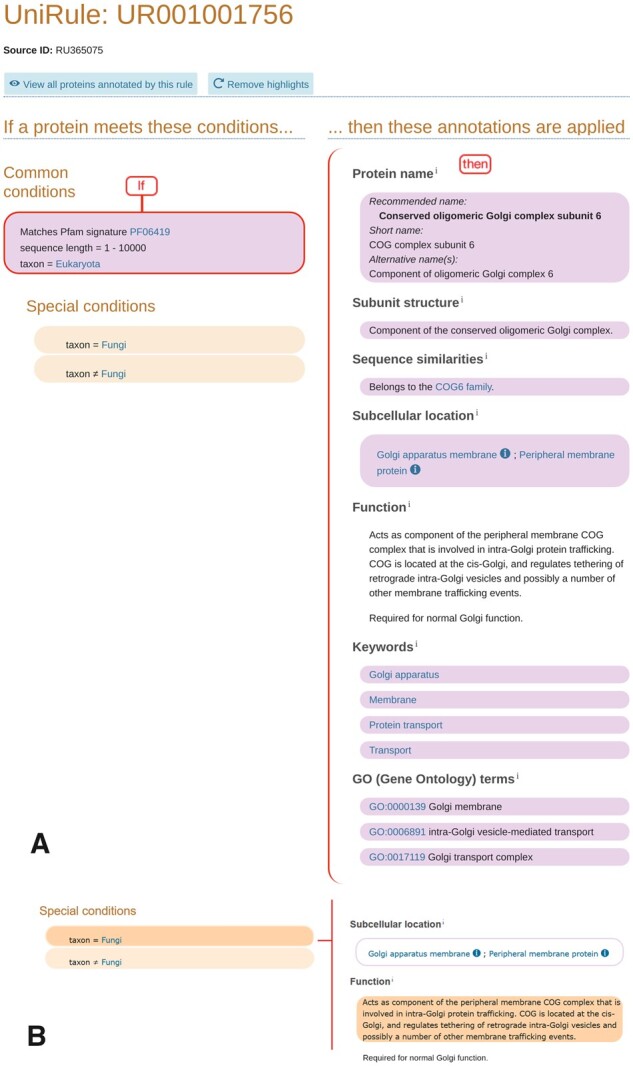
An example UniRule rule as displayed on the UniProt website. (**A**) UniRule display after the common conditions are selected (highlighted in lilac), showing the annotations (highlighted in lilac) that are applied to records meeting the conditions. (**B**) Part of the UniRule display after the first special condition, ‘taxon = Fungi’ is selected. The condition is highlighted in brown and the annotation that is added when this condition is met is also in brown. (https://www.uniprot.org/unirule/UR001001756)

To demonstrate how a rule is built, we focus on the ‘conserved oligomeric Golgi complex subunit 6’ that is part of the complex required for normal Golgi morphology and localization in eukaryotes. This protein belongs to the family COG6 for which the Pfam signature is PF06419. The representation on the UniProt website (at the time of writing) of the rule created starting with this signature is shown in Figure 1.

### 2.2 The structure of a rule

#### 2.2.1 Rule conditions

A condition is a constraint that a protein must satisfy to trigger propagation of the annotation from a rule. Some of the conditions make use of the main attributes of a reviewed UniProtKB record, which include taxonomy, InterPro matches, sequence length and phylogenetic information (where the protein is a member of an Ensembl GeneTree). Other conditions require a match to a particular amino acid sequence within the protein and make it possible to add annotation based on the presence of sequence features (see Section 2.2.4). This is useful in situations where InterPro signatures alone are not enough to specify detailed annotation at certain sequence locations (for instance, enzyme cofactor binding sites).

A condition requiring the presence of an InterPro signature is the normal starting point for preparing a rule. Currently the protein signatures used by UniRule are restricted to signatures from InterPro member databases (Mitchell *et al.*, 2019). However, the close working relationship between UniProt and InterPro means that signatures from member databases that are integrated into InterPro at each new database release are immediately available for use in UniRule. An InterPro signature that represents a family of proteins is generally used, and this may be combined with other InterPro signatures for domains to refine and restrict the rule to reviewed records with consistent annotation. Where protein signatures are not available to annotate a protein family, new HAMAP and PIRSF (Nikolskaya *et al.*, 2006) protein signatures (which are generated by members of the UniProt consortium) can be specifically created and incorporated into InterPro for use in UniRule. UniProt also works closely with the InterPro member database Pfam (El-Gebali *et al.*, 2019). When UniProt biocurators are reviewing proteins and adding them to the reviewed Swiss-Prot section of UniProtKB, a request can be made to have Pfam signatures specially created and made available to UniRule through InterPro. An example where this has occurred is Pfam: PF17073 (IPR031411) which is used in rule UR000212824 (https://www.uniprot.org/unirule/UR000212824).

Protein family databases differ in the intended scope of the family signatures they generate. For databases which aim for broad coverage (e.g. Pfam and PROSITE), the signatures can often be matched to proteins from archaea, bacteria and eukaryota. Other databases (e.g. PANTHER, TIGRFAMs, PIRSF, HAMAP) often generate signatures of very narrow scope, and the matches may be restricted to a limited taxonomic range. Whichever type of InterPro signature forms the starting point for a UniRule rule, the use of taxonomic conditions plays an important role in restricting the propagation of functional annotation to taxonomic groups for which the annotation is relevant.

Where the InterPro signature has limited taxonomic scope, the use of a taxonomic condition acts as a safety feature to avoid inappropriate annotation to taxa outside this scope. Length conditions can be added to ensure that the newly annotated protein falls within the known length distribution of the protein set. This helps to avoid the propagation of conflicting annotation, which can arise if there is an overlap between the sets of proteins annotated by different rules. For example UR001001756 (Fig. 1; https://www.uniprot.org/unirule/UR001001756) contains a length condition from 1 to 900, which reduces the number of unreviewed hits annotated by 4%, but enables the rule to avoid providing duplicate, and conflicting, annotation for a number of unreviewed records annotated by other rules. Length conditions also enable rules to avoid annotating fusion proteins.

#### 2.2.2 Rule annotations

Annotations are the information that the rule will propagate. These annotations are taken from expert reviewed entries (UniProtKB/Swiss-Prot) and are based on the published information available for the protein records. Properties such as protein name, general functional annotation, catalytic activity, pathway, GO terms and subcellular location are examples of possible annotation types. The use of controlled vocabularies or free text in annotations follows the current standards found in UniProtKB. There are certain controlled-vocabulary annotations in UniProtKB which are not suitable for propagation by UniRule. These include technical terms associated with disease (as these are organism specific), and keywords from the categories ‘Technical term’ (e.g. Complete proteome) and ‘Coding sequence diversity’ (e.g. Alternative splicing) (https://www.uniprot.org/keywords/).

#### 2.2.3 Organization of rule conditions and annotations into sets

**Fig. 2.**
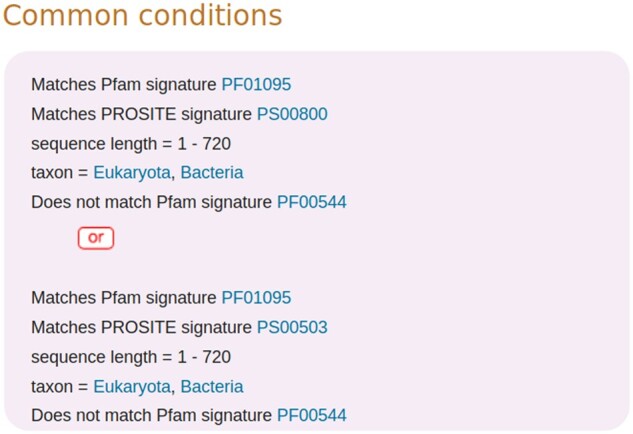
The common conditions in a UniRule rule. Rule UR000000256 contains two sets of conditions that make up the common conditions. Within each set, the conditions are connected by the logical operator ‘AND’, while the two sets are connected by the logical operator ‘OR’

The conditions within a rule are organized into sets where the logical operator connecting the different conditions within a set is ‘AND’. One or more of these sets of conditions are then combined with the logical operator ‘OR’ to make up the ‘common conditions’ which define the overall scope of the rule. The rule UR000000256 (Fig. 2) is an example where two sets of conditions are used to define the common conditions.

A rule may contain further sets of conditions known as ‘special conditions’ that are used to define particular subgroups of the main set of records. This organizational structure allows one set of annotations to be given to all the records that meet the common conditions, and additional annotation to be given to a particular subgroup of records that also meet the special conditions. In this way, a high level of control is exercised over the annotation that different records receive. Figure 1 illustrates how this works in practice, with each of the two panels (A and B) presenting a partial view of the same rule as displayed on the UniProt website. In each case the conditions are shown on the left side of the rule diagram, and the annotations are on the right. In this example there is only one set of conditions within the set of common conditions. In Figure 1A, the common conditions have been selected (by clicking on them in the web page) and as a consequence, both the common conditions and the annotations that are propagated to records that meet the common conditions, are highlighted in lilac. So, for instance, unreviewed records from the eukaryota that have hits for PF06419 and are up to 900 residues long receive the subcellular location annotation ‘Golgi apparatus membrane’ and ‘Peripheral membrane protein’. However, annotation for function is not added to records on the basis of the common conditions alone and is not highlighted. Figure 1B shows the appearance of the webpage after the special condition ‘taxon = Fungi’ has been clicked. The special condition is highlighted in brown, as also is the annotation for function that is applied if both the common conditions and this special condition are met. If the special condition ‘taxon ≠ Fungi’ had been selected instead, then the function annotation highlighted would have been: ‘Required for normal Golgi function’.

#### 2.2.4 Specifying sequence features in special conditions

**Fig. 3.**
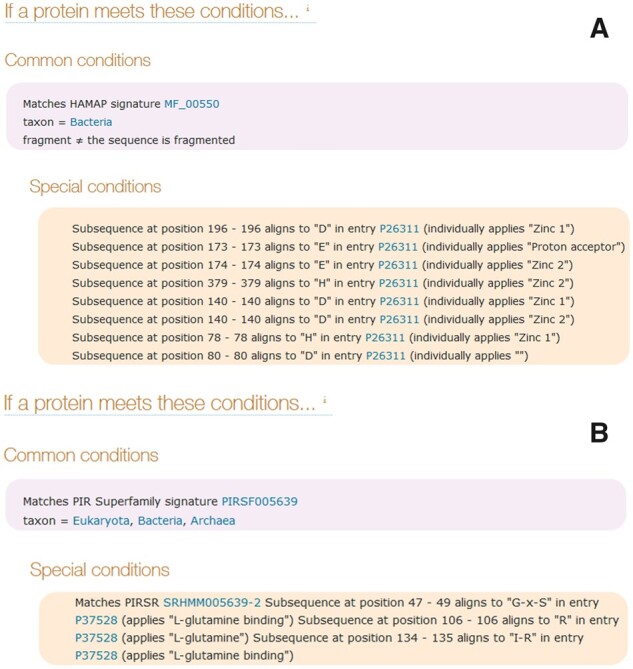
Special conditions that provide site-specific annotation in protein sequences. (**A**) The conditions from a HAMAP rule UR000101617. (**B**) The conditions from a PIRSR rule UR001165955

UniRule rules may also contain special conditions that provide for more fine-grained, sequence-specific information such as the location of active sites, post-translationally modified residues and residues of functional importance. Special conditions of this type are an important component of rules incorporated into UniRule from HAMAP (Pedruzzi *et al.*, 2015) and PIRSR (Chen *et al.*, 2019). Where this type of condition is used, a template sequence is nominated and a list of patterns is provided, together with their location in the template sequence and the annotation associated with the presence of the pattern. All the target records that meet the common conditions are aligned to the template sequence and evaluated for the presence of the patterns in the target sequence at the corresponding locations. Where there is a match, the annotation is added to the target record. Examples of the special conditions which are used to provide sequence specific annotation in rules incorporated from HAMAP and PIRSR are shown in Figure 3.

#### 2.2.5 Evidence codes used by UniRule

**Fig. 4.**
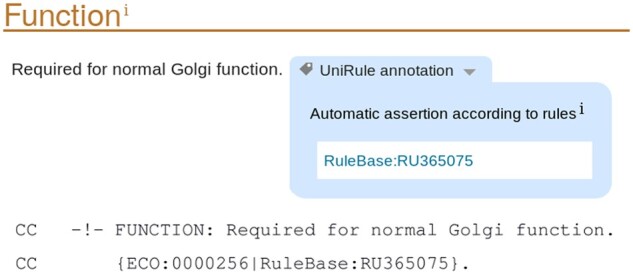
The representation in the UniProt website of evidence codes for annotation propagated by UniRule UR001001756. Above: Web page display; Below: Text version of the protein record

Whenever annotation is added to an unreviewed record as a result of UniRule, an evidence code is included from the Evidence and Conclusion Ontology (Giglio *et al.*, 2019), together with the identifier for the rule that gave rise to the annotation. UniRule annotation uses the code ECO:0000256, ‘match to sequence model evidence used in automatic assertion’. For example the function annotation propagated by UR001001756 shown in Figure 1 is displayed in the website view of the record and in the text version of the protein record as shown in Figure 4.

### 2.3 Rule updates and quality maintenance

#### 2.3.1 Quality control during rule creation

The two main quality criteria against which a new rule needs to be judged are: (i) do the reviewed proteins identified by the rule conditions form a distinct set which is biologically coherent and consistently annotated; (ii) is there overlap between the new rule and existing rules that will lead to conflicting annotation being propagated to individual unreviewed entries.

To help with the first quality issue, the UniRule system provides rule curators with different statistical measures. These include the true-positive (TP) and false-positive (FP) numbers for each of the annotations in the set of reviewed records defined by the rule conditions. TP and FP are used in their standard statistical sense, where TP refers to the number of records meeting the conditions which also contain the annotation, and FP refers to the number of records meeting the conditions which do not contain the annotation. False-negative (FN) values (records that contain the annotation but are not in the set defined by the conditions) are also shown. To be accepted into the UniRule pipeline a rule must have a confidence level (TP/(TP + FP)) of at least 0.95 for all the annotations it contains. However, curators do have the option to exclude specific annotations in individual records from statistical calculation. A common reason for exclusion is semantic variation between annotations which is not biologically relevant but that arises because some UniProtKB annotation fields contain free text.

To help curators address the question of overlap between different rules, the statistical data for each rule includes a link to the list of unreviewed entries that will be annotated by a rule, and a link to all the rules with which there is an overlap, together with links to the individual unreviewed entries that are affected by the overlap. These features allow curators to create new rules that extend the coverage of the database without repeating or conflicting with existing rules.

#### 2.3.2 Correcting and updating between UniProt releases

InterPro protein signatures, and the reviewed UniProtKB/Swiss-Prot records that form the basis of UniRule, are not static, but are each revised and updated regularly. At each release of UniProtKB, statistical checks are carried out on the rules in UniRule to identify instances where a rule contains an InterPro signature that has been revised or withdrawn, or where the reviewed UniProtKB records supporting a rule no longer contain consistent annotation—which may be caused by record updates or additions of new records. These issues are then resolved by curators by either revising the conditions and annotations of a rule, or, if appropriate, by updating the content of the UniProtKB/Swiss-Prot records on which the rule is based. If no satisfactory resolution is possible, the rule is taken out of production, but can be re-applied in a later release if the problem is resolved.

## 3 Results

### 3.1 Annotation coverage and annotation depth

Coverage and depth are two important measures of the extent of predicted annotation within UniProtKB. Coverage refers to the proportion of records that receive any annotation and to the distribution of these records across the taxonomic space. Depth refers to the extent of annotation within the records annotated.

For release 2020_01 of UniProtKB, UniRule provided annotation for 53 million records out of a total of 178 million in the unreviewed UniProtKB/TrEMBL section. UniRule provided annotation to 38, 21, 30 and 30%, respectively, of the records for viruses, archaea, bacteria and eukaryota. Annotation coverage was also fairly consistent across the taxonomic subdivisions of each of these domains. For example coverage within the main classes of bacteria ranged from 23 to 34%. Within the eukaryota, coverage for fungi, metazoa and viridiplantae was 22, 38 and 29%, respectively.

The total number of individual annotations provided by UniRule in UniProtKB release 2020_01 was 722 million. A simple measure of the depth of annotation is the extent to which individual records receive annotations in each of four main categories: (i) Protein name; (ii) Comment (similarity, function, subunit, etc.); (iii) Sequence Feature and (iv) Keyword. For release 2020_01, taking all the records that received some annotation as 100%, 32% received annotation for all four annotation categories; 12% received annotation for three categories; 38% received annotation for two categories and 15% received annotation for only one category. Certain categories are prioritized in annotation, for instance providing a protein name, thus annotation of one category only can still provide useful information on the nature of the protein. However, providing depth of annotation as well as a high degree of coverage is a priority for UniProtKB automated annotation.

Coverage resulting from UniRule of the unreviewed entries that make up UniProtKB/TrEMBL is regularly reviewed by UniProt. Key priorities are to achieve broad annotation across all taxa, and particularly to propagate annotation for enzyme activity to unreviewed entries.

### 3.2 Benchmarking

#### 3.2.1 Benchmark dataset

We collected protein records from the beginning of 2014 to the end of 2019 that were annotated by UniRule rules and in the next UniProtKB release were then manually reviewed. In total, 3575 records were collected with an average of 595 per year.

#### 3.2.2 Annotations quality

We estimate the performance of UniRule in terms of quality through three measures:

*Precision* P=TP/(TP+FP): to measure the quality of generated annotations compared with manual review.

*Recall* R=TP/(TP+FN): to measure the sensitivity of UniRule i.e. its ability to generate rules for annotations yet to be revealed by manual review.

$F_{\beta }$ measure (or simply *F* measure) $=(\beta^{2}+1)P*R/(\beta^{2}*P+R)$:

*β* is a parameter that controls the balance between *P* and *R*. When *β *= 1, $F_{\beta }$ measure becomes equivalent to the harmonic mean of *P* and *R* [i.e. *F*_1_ measure=2 * *P* * *R*/(*P*+*R*)]. If $\beta >1,\;F_{\beta }$ measure becomes more recall-oriented and if β<1, it becomes more precision-oriented. Formally, *β* is defined as follows:

β=R/P

**Fig. 5.**
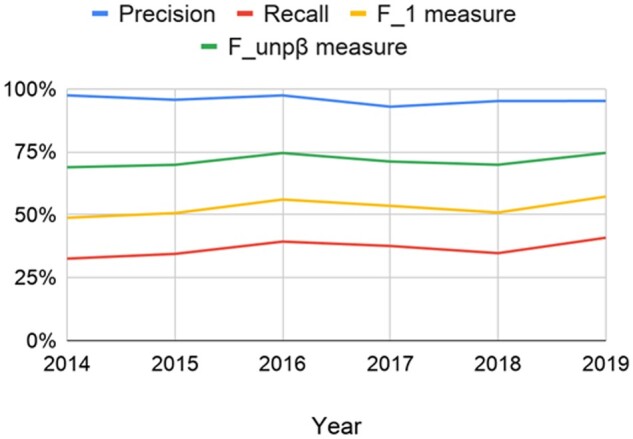
Evolution of annotation quality from 2014 to 2019

In UniRule, quality is precision-oriented. Indeed, for a rule to be used to annotate entries, precision *P* and recall *R* must respectively be within [0.95, 1] and ]0, 1]. Hence *β* can be estimated as:

β=average(R)/average(P)=((0.95+1)/2)/((0+1)/2)=0.51 ; we term this value as unpβ. Figure 5 illustrates the evolution of annotation quality from 2014 to 2019 through four curves: *P* curve, *R* curve, *F*_1_ curve and $F_{\text{unp}\beta }$ curve. It shows that precision has been kept high over this time, and is generally close to 100%, i.e. the quasi totality of annotations predicted by UniRule were later revealed by manual review as correct. Recall increased from 2014 to 2019 by 8% i.e. UniRule was becoming more sensitive and able to predict a greater number of annotations. *F*_1_ and $F_{\text{unp}\beta }$ measures also improved respectively by 8 and 6%.

## 4 Implementation

### 4.1 Integration of UniRule into the UniProtKB pipeline

**Fig. 6.**
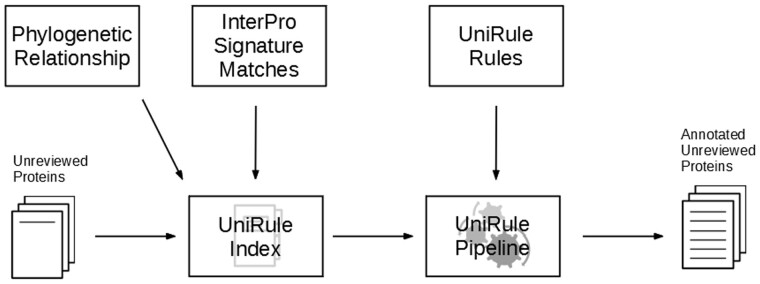
Diagram of the pipeline that applies UniRule annotations to UniProtKB. See the main text for a description of the pipeline

With each release of UniProtKB/TrEMBL new unreviewed proteins are added and the attributes of existing proteins (which determine whether a record meets the conditions of a rule) are updated. Therefore, the predictions for all unreviewed proteins have to be re-computed using the latest version of the UniRule rules and the latest data for protein attributes. Taking into account the huge number of unreviewed proteins and its exponential growth in time, this procedure needs to be highly efficient and scalable to fit into the UniProtKB production cycle. To satisfy these requirements we created the UniRule Pipeline, a software that generates functional annotations for unreviewed proteins from their attributes and a set of rules. The rules developed by the different UniRule collaborators (RuleBase, HAMAP, PIRNR, PIRSR) are all expressed in a common XML format for ease of handling and error checking. The UniRule Pipeline also uses a Lucene index based on protein attributes, which we refer to as the UniRule Index. This index combines all necessary protein attributes and allows evaluation of the conditions of a rule against the set of unreviewed proteins in an efficient and scalable way. The application process of all UniRule rules to the ∼180 million unreviewed proteins in UniProtKB of release 2020_01 required a total runtime of ∼1.5 h on 100 CPU cores. The UniRule pipeline is summarized in Figure 6.

### 4.2 Availability

Currently the simplest way of accessing UniRule data is through the UniProt website (https://www.uniprot.org/unirule/). The conditions, annotations and the relationship between them are displayed in a graphical format (as in Fig. 1). The rule display also contains links to the reviewed entries on which the rule is based, and the unreviewed entries which receive the annotation.

The UniProt Consortium is committed to making the rules available in a format which enables researchers to annotate their own protein sequences independently of UniProt, and which enables independent rule generators to contribute their efforts to the further annotation of UniProt. There are three components required to run UniRule rules on a user-generated protein dataset. First, the rules need to be obtained from the EMBL-EBI ftp site (ftp.ebi.ac.uk/pub/contrib/UniProt/UniFIRE/rules/). The rules are held in an XML format (UniProt Rule Markup Language, URML) that contains both the conditions and annotations, and the logic used in the application of the rule. Second, the user needs to run InterProScan (Jones *et al.*, 2014) to obtain the InterPro matches to their data. Third, the rules and the InterProScan output are combined to generate the annotation, using software developed at UniProt (Uniprot Functional annotation Inference Rule Engine, UniFIRE). UniFIRE is available from https://gitlab.ebi.ac.uk/uniprot-public/unifire and is based on the Business Rules Management System, Drools. The steps to annotate third party data are under continuous development and are subject to change as they are being improved and developed. A webinar that presents the current steps is available at: https://www.youtube.com/watch?v=_7wuufRp-GM.

To simplify the process of setting up UniFIRE, a Docker image has been made available to download. This image contains all the required dependencies and makes it possible to start a generic UniFIRE workflow with a single command. Documentation that explains the different ways to run UniFIRE is available at: https://gitlab.ebi.ac.uk/uniprot-public/unifire/blob/master/README.md.

The application of the Docker image to a complete bacterial proteome of 4500 proteins, using an Intel Core i5-4690 CPU with 4 cores, requires a runtime of 6 h. Almost all of this runtime (98%) is needed for the InterProScan procedure, while the UniFIRE software requires a runtime of 6 min. InterProScan can potentially be speeded up significantly using the option to use the lookup of pre-calculated matches provided by InterProScan.

## 5 Discussion

UniRule takes advantage of the quality and consistency of the data in the expertly reviewed and annotated section of UniProtKB (UniProtKB/Swiss-Prot) and propagates annotation to the very large number of predicted proteins in the unreviewed section of UniProtKB (UniProtKB/TrEMBL). The software pipeline that performs this task integrates the HAMAP (Pedruzzi *et al.*, 2015), PIRNR/PIRSR (Chen *et al.*, 2019) and RuleBase annotation systems developed by the UniProt Consortium members into one workflow. UniRule annotates the unreviewed records in UniProtKB in a way that is closely coordinated with the release cycle of UniProtKB. The UniRule system also includes a number of statistical checks which ensure that UniRule curators are able to correct and update the rules at each UniProtKB database release to respond to changes in the underlying data on which the rules are based. Recent developments have made the rules available to the scientific community together with software that enables third party protein sequences to be annotated. This extends the role of UniRule beyond its use as an internal system to augment annotation in UniProtKB, and makes it a powerful means of transferring the knowledge and information accumulated in the reviewed section of UniProtKB to the protein research community.

## UniProt Consortium

European Bioinformatics Institute^1^: Alex Bateman, Michele Magrane, Maria J. Martin, Sandra Orchard, Emily Bowler, Ramona Britto, Hema Bye-A-Jee, Paul Denny, Penelope Garmiri, Emma Hatton-Ellis, Antonia Lock, Yvonne Lussi, Alistair MacDougall, Elena Speretta, Nidhi Tyagi, Kate Warner, Rossana Zaru, Shadab Ahmed, Emanuele Alpi, Austra Cukura, ThankGod Ebenezer, Leonardo Gonzales, Abdulrahman Hussein, Alexandr Ignatchenko, Giuseppe Insana, Rizwan Ishtiaq, Vishal Joshi, Dushyanth Jyothi, Aurelien Luciani, Jie Luo, Mahdi Mahmoudy, Katie Moulang, Andrew Nightingale, Joseph Sampson, Sangya Pundir, Guoying Qi, Daniel Rice, Rabie Saidi, Edward Turner, Preethi Vasudev, Xavier Watkins, Hermann Zellner. SIB Swiss Institute of Bioinformatics^3^: Alan Bridge, Lionel Breuza, Elisabeth Coudert , Damien Lieberherr, Ivo Pedruzzi, Sylvain Poux, Manuela Pruess, Nicole Redaschi, Lucila Aimo, Ghislaine Argoud-Puy, Andrea Auchincloss, Kristian Axelsen, Emmanuel Boutet, Cristina Casals-Casas, Anne Estreicher, Maria Livia Famiglietti, Marc Feuermann, Arnaud Gos, Nadine Gruaz, Chantal Hulo, Nevila Hyka-Nouspikel, Florence Jungo, Philippe Le Mercier, Patrick Masson, Anne Morgat, Sandrine Pilbout, Catherine Rivoire, Christian Sigrist, Shyamala Sundaram, Parit Bansal, Delphine Baratin, Teresa Batista Neto, Jerven Bolleman, Beatrice Cuche, Edouard De Castro, Elisabeth Gasteiger, Sebastien Gehant, Arnaud Kerhornou, Thierry Lombardot, Monica Pozzato. Protein Information Resource^4^: Cathy Wu, Cecilia Arighi, Hongzhan Huang, Peter McGarvey, Darren Natale, Shriya Raj, John S. Garavelli, Kati Laiho, Lucille Pourcel, Karen Ross, C. R. Vinayaka, Qinghua Wang, Lai-Su Yeh, Leslie Arminski, Chuming Chen, Yongxing Chen, Aurelian Luciani, Alok Mishra, Yuqi Wang, Jian Zhang.

## Funding

This work was supported by the National Eye Institute (NEI), National Human Genome Research Institute (NHGRI), National Heart, Lung, and Blood Institute (NHLBI), National Institute on Aging (NIA), National Institute of Allergy and Infectious Diseases (NIAID), National Institute of Diabetes and Digestive and Kidney Diseases (NIDDK), National Institute of General Medical Sciences (NIGMS), National Institute of Mental Health (NIMH) and National Cancer Institute (NCI) of the National Institutes of Health (NIH) [U24HG007822]; British Heart Foundation [RG/13/5/30112]; Parkinson’s Disease United Kingdom [PDUK GO grant G-1307]; Alzheimer’s Research UK [ARUK-NAS2017A-1]; National Science Foundation (NSF) [DBI-1062520]; NIH [GO grant U41HG02273]; National Institute of General Medical Sciences [R01GM080646, P20GM103446, G08LM010720]; Open Targets; European Molecular Biology Laboratory (EMBL) core funds; and Swiss Federal Government through the State Secretariat for Education, Research and Innovation (SERI).

*Conflict of Interest*: none declared.
