# UniRule: a unified rule resource for automatic annotation in the UniProt Knowledgebase

**DOI:** 10.1093/bioinformatics/btaa663

**Published:** 2021-03-17

**Authors:** Alistair MacDougall, Vladimir Volynkin, Rabie Saidi, Diego Poggioli, Hermann Zellner, Emma Hatton-Ellis, Vishal Joshi, Claire O’Donovan, Sandra Orchard, Andrea H Auchincloss, Delphine Baratin, Jerven Bolleman, Elisabeth Coudert, Edouard de Castro, Chantal Hulo, Patrick Masson, Ivo Pedruzzi, Catherine Rivoire, Cecilia Arighi, Qinghua Wang, Chuming Chen, Hongzhan Huang, John Garavelli, C R Vinayaka, Lai-Su Yeh, Darren A Natale, Kati Laiho, Maria-Jesus Martin, Alexandre Renaux, Klemens Pichler, and The UniProt Consortium

*Bioinformatics* (2020) doi: 10.1093/bioinformatics/btaa485

In the above article, co-authors Alexandre Renaux and Klemens Pichler were initially omitted. This has now been corrected.

